# Kin and Non-Kin Connected Plants Benefit More Than Disconnected Kin and Non-Kin Plants under Nutrient-Competitive Environments

**DOI:** 10.3390/plants12030487

**Published:** 2023-01-20

**Authors:** Jan Sher, Farkhanda Bibi, Gul Jan, Kyle W. Tomlinson, Asma Ayaz, Wajid Zaman

**Affiliations:** 1 Center for Integrative Conservation, Xishuangbanna Tropical Botanical Garden Chinese Academy of Sciences, Mengla 666303, China; 2 University of Chinese Academy of Sciences, No. 19A Yuquan Road, Beijing 100049, China; 3 Department of Botany, Garden Campus, Abdul Wali Khan University Mardan, Mardan 23200, Pakistan; 4 State Key Laboratory of Biocatalysis and Enzyme Engineering, School of Life Sciences, Hubei University, Wuhan 430062, China; 5 Department of Life Sciences, Yeungnam University, Gyeongsan 38541, Republic of Korea

**Keywords:** biomass accumulation, biomass allocation, *Chenopodium quinoa*, kinship, niche partitioning, nutrients, root connectivity

## Abstract

In the natural environment, plants grow and interact with both conspecific and heterospecific neighbours under different environmental conditions. In this study, we tested whether *Chenopodium quinoa* Willd genotypes differ in growth performance when grown with kin and non-kin under nutrient limitation in pot partitioning treatments. Biomass accumulation, allocation, organ efficiency, and specific leaf area were measured at the end of the experiment. Response variables were differentially impacted by kinship, fertility, and barrier. Total dry mass, shoot dry mass, and root and stem allocation were greater for plants grown with kin in connected pots than with non-kin in connected pots across the nutrient treatments. Kin connected and disconnected plants had a greater specific root length, specific stem length, and average leaf mass than non-kin connected and disconnected plants. Non-kin connected and disconnected plants had greater LAR and SLA than kin connected and disconnected plants under low- and high-nutrient treatments. Plants always grew better in the presence of their kin than non-kin. These results conclude that quinoa plant production benefits from planting closely related individuals under both high- and low-nutrient conditions.

## 1. Introduction

Root communication between plants for soil resources is a common aspect in plants [1,2,3,4] which contributes to the structure and function of ecosystems [5,6,7]. The physical connection between plants through belowground roots or aboveground shoots provides a mechanism for many types of coordinated growth. This type of coordination between plants can be for nutrient resources, for cooperation with each other, and for the transfer of different signals through roots or aboveground when plants are in stressful conditions [8,9,10,11]. Plants can detect whether a neighbour is attacked by herbivores or signalled by VOCs [12], or experiences environmental stress such as nutrient, light, or water stress [10,13,14,15]. The first study on plant response to nutrients and neighbours was conducted on *Glycine max* L., using a split root experiment design with two individuals connected or disconnected by a partition [16]. The findings showed that soybean plants allocated 85% of their biomass to roots when interacting with neighbours and only 30% to roots when grown alone. 

Plants are more likely to stay with closely related individuals due to inbreeding, seed dispersal, and asexual reproduction [17,18]. Research shows that plants can discriminate their own roots and also when they grow near their neighbour kin or non-kin plants [19,20]. Kinship interaction is confirmed in many annual plant species, but the main mechanisms of plant kinship interactions are poorly understood. Hamilton’s rule (Kin selection) [21] predicts that genetically related individuals (kin) cooperate with each other because they belong to the same parents and may show altruistic behaviour to reduce group fitness [18]. Several studies have shown that plants perform better when grown with close relatives [22,23,24]. Some studies have reported that kin/siblings reduced their own fitness when they have grown with their kin/siblings [25,26]. Research on the annual plant *Cakile edentula* [27] found that individuals reduced their belowground root competition (i.e., reduced allocation to root, and reduced root length) when grown with their kin neighbours, relative to non-kin. Research on *Impatiens pallida* [13] found that *I. pallida* increased their aboveground competition in the presence of non-kin neighbours, relative to kin neighbours. Conversely, niche partitioning from ecological theory predicts that kin/siblings are more competitive than non-kin/non-siblings because more closely related individuals share more similar niches [24,28,29]. The controversy between these two predictions was tested in this study.

Nutrient deficiency can have a significant impact on agriculture, resulting in reduced overall plant growth performance, crop yield, and plant quality [30,31]. The study aims to understand how interactions between plants and their neighbour kin or non-kin can be applied to improve our understanding of the functioning of crop systems and how this knowledge can lead to the design of more optimal plant types and more optimal crop management. 

In this study we considered growth performance of different genotypes of *Chenopodium quinoa* Willd. (Amaranthaceae), commonly known as quinoa, when grown in kin and non-kin combinations under low- and high-nutrient conditions, with or without root connection (barrier). Quinoa is a stress tolerant crop, able to cope in both dryland and saline conditions [32]. We asked the following questions: (1) Do connected plants increase competition or cooperation between kin and non-kin plants relative to disconnected plants? (2) How do these belowground interactions alter biomass accumulation, allocation, and morphology in kin versus non-kin combinations? We hypothesized that (1) root–root competition is stronger in kin plants than non-kin because closely related individuals (kin) use similar resources and compete for more similar resources than non-kin, causing non-kin plants to have greater aboveground biomass, i.e., total dry mass and organ mass fraction, and fitness than kin plants. (2) Belowground competition is stronger under low-nutrient soil relative to high-nutrient soil because under nutrient-limited soil, plants elongate their roots more to access more nutrient resources and, as a result, plants increase their belowground biomass allocation for root and root length, i.e., root mass fraction (RMF) and specific root length (SRL). (3) Disconnecting plants eliminates this difference in plants grown next to kin and non-kin because competition for shared resources is removed. 

## 2. Results

A summary of the best models selected for each response variable is provided in Table 1. Most of the response variables did show significant variation in response to kinship, fertility, and barrier. Total dry mass (TDM) and shoot dry mass (SDM) were affected by three-way interaction of kinship, fertility, and barrier. Leaf mass fraction (LMF) and stem mass fraction (SMF) were only affected by fertility. Root mass fraction (RMF) was affected by kinship, fertility, and barrier, while specific root length (SRL), specific stem length (SSL), and average leaf mass (ALM) were affected by kinship and barrier. Leaf area ratio (LAR) and specific leaf area (SLA) were both affected by three-way interactions between kinship, fertility, and barrier.

### 2.1. Response of Biomass Accumulation

**Figure 1:**
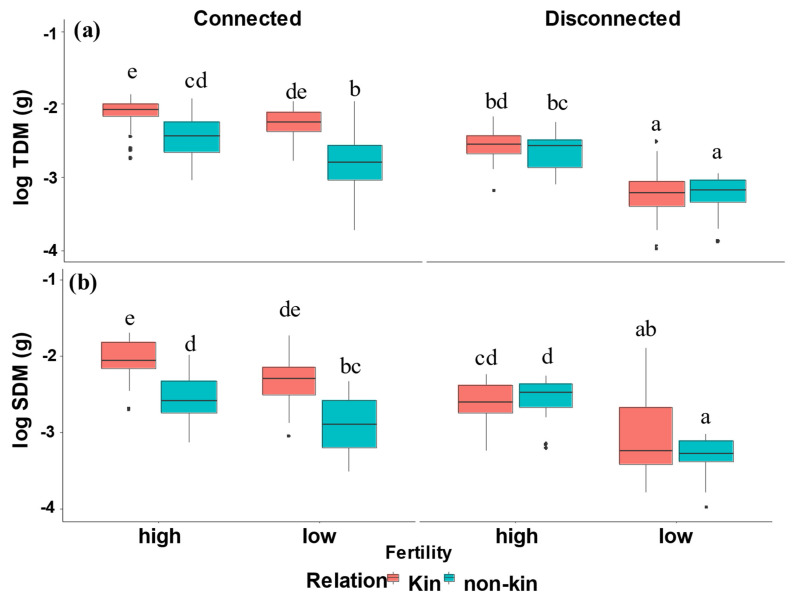
(**a**) Total dry mass, (**b**) Shoot dry mass of kin and non-kin under low nutrients versus high nutrients, connected versus disconnected. Common letters within each panel indicate no statistically significant difference among the treatment pairs in that panel (based on Tukey HSD tests).

Total dry mass (TDM) and shoot dry mass (SDM) were impacted by three-way interactions (kinship, fertility, and barrier) (Table 1, Figure 1). Kin connected plants accumulated greater biomass than non-kin connected plants under low- and high-nutrient soil. Disconnected kin and non-kin plants did not differ from one another, but growth was greater under high nutrients. 

### 2.2. Response of Biomass Allocation 

**Figure 2:**
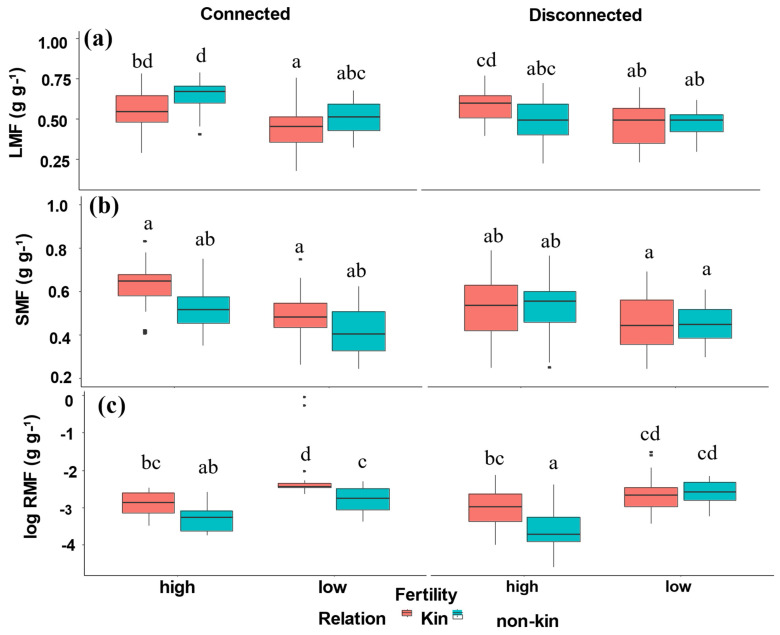
(**a**) Leaf mass fraction (LMF), (**b**) Stem mass fraction (SMF), and (**c**) Root mass fraction (RMF) of kin and non-kin in all treatment combinations including low- versus high-nutrient connected and low- versus high-nutrient disconnected treatments. Common letters within each panel indicate no statistically significant difference among the treatment pairs in that panel (based on Tukey HSD tests).

Allocation to leaves (LMF) and allocation to stem (SMF) were significantly impacted by nutrient levels (fertility) (Table 1, Figure 2). Non-kin connected plants had a greater LMF than kin under low- and high-nutrient soil, while there were no significant differences between disconnected kin and non-kin plants. Kin connected plants allocated more biomass into the shoot fraction (SMF) than non-kin plants independently from soil nutrient concentrations, while no significant differences were observed in disconnected kin and non-kin plants. 

Allocation to roots (RMF) was significantly impacted by all treatments in an additive manner (Table 1, Figure 2). Kin connected plants allocated greater RMF than non-kin connected plants under both low- and high-nutrient soil. Kin disconnected plants allocated greater RMF than non-kin disconnected plants in high-nutrient soil, while there were no differences observed under low-nutrient soil for disconnected kin and non-kin plants.

### 2.3. Organ Efficiency Parameters 

**Figure 3:**
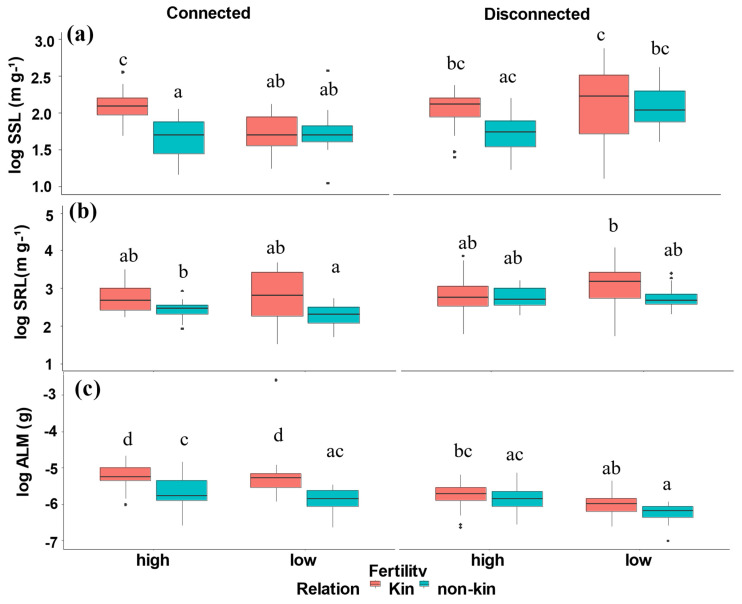
(**a**) Specific stem length (SSL), (**b**) Specific root length (SRL), and (**c**) Average leaf mass (ALM) of kin and non-kin in all treatment combinations including low- versus high-nutrient connected and low- versus high-nutrient disconnected treatment. Common letters within each panel indicate no statistically significant difference among the treatment pairs in that panel (based on Tukey HSD tests).

Specific stem length (SSL) and specific root length (SRL) were impacted by relation and barrier in an additive manner, while average leaf mass (ALM) was impacted by three-way interaction among all treatments (Table 1, Figure 3). Kin connected and disconnected plants had greater SSL, SRL, and ALM than non-kin connected and disconnected plants. 

### 2.4. Response of Leaf Area Ratio (LAR) and Specific Leaf Area (SLA)

**Figure 4:**
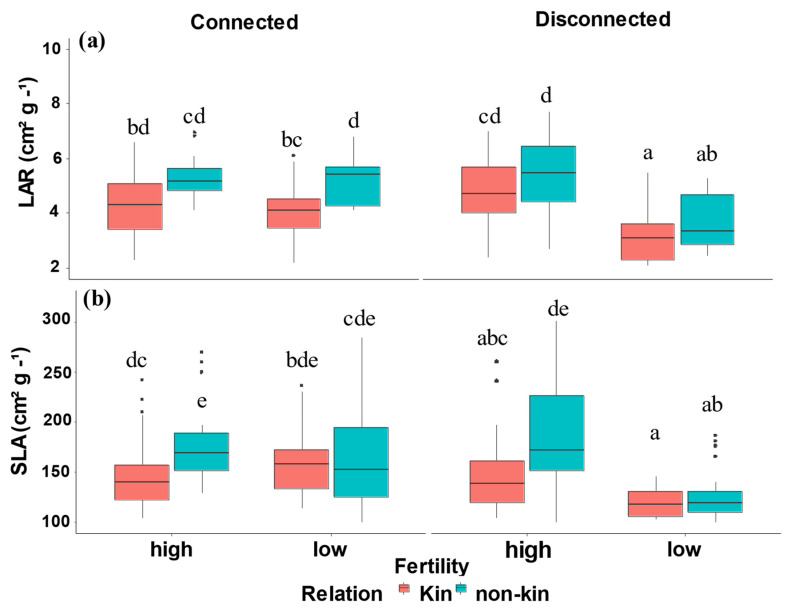
(**a**) Leaf area ratio (LAR) and (**b**) Specific leaf area (SLA) in all treatment combinations of kin and non-kin including low- versus high-nutrient connected and low- versus high-nutrient disconnected treatments. Common letters within each panel indicate no statistically significant difference among the treatment pairs in that panel (based on Tukey HSD tests).

Leaf area ratio and specific leaf area were significantly impacted by three-way interactions among all treatments (relation, fertility, and barrier) (Table 1, Figure 4). Non-kin connected and disconnected plants had greater LAR and SLA than kin connected and disconnected plants under low- and high-nutrient treatments.

### 2.5. Multivariate Analysis of Traits across Individuals

**Figure 5:**
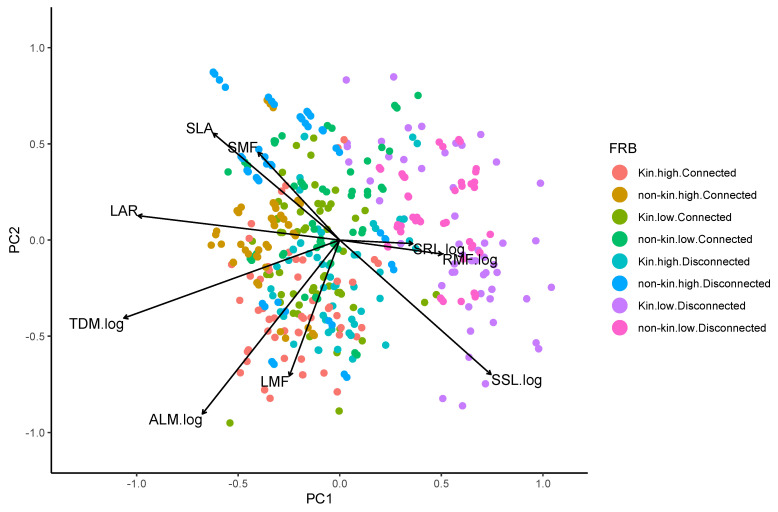
Principal components analysis of the individual × trait value data. The first and second principal components account for 24.5% and 18.4% of the total inertia respectively. Colours indicate treatment combinations for individual plants in the experiment. The trait distributions indicate that the first axis represents a trade-off between allocation to roots on the right versus allocation to light capturing surface and total biomass accumulation on the left. Trait spread along the second axis suggests a tradeoff between total allocation to leaves on the bottom and allocation to stems on the top.

Principal components analysis (PCA) of the plant traits measured (barring SDM, which was highly positively correlated with TDM, r = 0.86; all other correlations were < 0.65) indicated that separation along the first principal component (PCA1) was significantly related to fertility treatment (r^2^ = 0.166) and barrier treatment (r^2^ = 0.146), and separation along the second principal component (PCA2) was significantly related to relation treatment (r^2^ = 0.076) (Figure 5). Individuals growing in disconnected treatments or low-nutrient treatments were associated with higher SSL, RMF, and SRL, whereas individuals growing in connected treatments or high-nutrient treatments were associated with greater total mass and leaf area per mass surfaces (LAR, ALM, and SLA). Kin individuals tended to have greater allocation to shoot components than non-kin individuals (LMF, SSL, and ALM). 

## 3. Discussion

Here, we tested how quinoa plants grow and perform when they grow with their connected or disconnected neighbour, kin or non-kin, defined by the same or different genotypes, under different nutrient availability. We found that genotypes significantly increased their biomass when they grew with their kin neighbours relative to non-kin neighbours in connected treatments, whereas they had no differences in biomass in disconnected treatments. This confirms that quinoa plants grow differently with closely related versus distantly related neighbours. In general, low fertility increased growth differences between plants grown with kin and non-kin (Figure 1). 

### 3.1. Biomass Accumulation and Allocation of Connected Plants to Soil Nutrient and Kinship 

Belowground interaction among plants may affect aboveground biomass in nutrient-competitive environments. Limited nutrient availability influences plant biomass production [33,34]. Overall, we found that total dry mass and shoot dry mass were greater for the kin connected plants than non-kin connected plants (Table 1, Figure 1), and the differences were greater under high nutrients relative to low nutrients. The results did not support our expectation; we predicted that non-kin should accumulate greater plant dry mass compared to kin, because kin plants should be subject to stronger competition for available resources belowground due to their shared niches. This suggests that quinoa plants receive fitness benefits from growing with kin [21,24,35]. Previous studies found that nutrient availability altered the strength of kin interactions, with competition amongst non-kin being stronger at low nutrient availability [36]. 

Biomass allocation to organs is a key component of plant life history and plays an important role in the trade-off between resource acquisition and resource utilization [37,38,39]. Plants can exhibit plasticity and adjust the distribution of their organs and systems to meet available resources [39]. Nutrient availability is one of the important factors driving changes in biomass allocation [38]. Overall, we found a significant difference in biomass allocation between kin and non-kin connected plants (Table 1, Figure 2). Allocation to leaves (LMF) was greater in connected non-kin than in connected kin plants, suggesting that non-kin connected plants compete more for the aboveground resources such as light and space [13]. Taken together with the results in LAR and SLA, the patterns suggest that non-kin compete more aboveground than belowground. Increased allocation to leaf or stem can indicate increased competition for light [39,40], but it may also indicate increased performance, such that greater allocation to stem could lay the foundation for increased seed crop [41]. Given that quinoa grows in dry environments at high elevations, where water and nutrient stress may be more important selective constraints than light, it seems unlikely that quinoa would possess phenotypic plasticity to respond to light competition [42,43], but would possess root plasticity to respond to water and nutrient supply [44]. 

### 3.2. Response of Leaf Area Ratio (LAR) and Specific Leaf Area (SLA)

Plants can adapt to different light and nutrient supply conditions, presumably to overcome resource limitations [45,46]. An important variable in their adaptation is the ratio of leaf area to root length, which is based on the allocation of plant biomass and organ morphology [39,45,47]. Specific Leaf Area (SLA) and Leaf Area Ratio (LAR) are important leaf characteristics because they represent the ratio of the leaf’s light-receiving surface per unit dry weight investment [45,48]. Overall, our results showed that non-kin plants and disconnected plants have a higher SLA and LAR relative to kin plants and connected plants (Table 1, Figure 4). Taken together with the results of LMF, the LAR and SLA of non-kin plants reflect an increase in mutual shading of the plants compared to kin. When nutrients level increased, LAR and SLA also increased, which can be interpreted as a plant growth strategy to reduce investment under the soil to generate more biomass in high-nutrient soils [35,49]. Kin plants had lower LAR and SLA, but that did not compromise for their total dry mass. Kin plants managed to generate greater biomass than non-kin while reducing LAR and SLA, resulting in less leaf overlap and shading, thereby increasing light-harvesting efficiency [50]. These patterns suggest quinoa plants can recognise their neighbor relatedness and grow in manner that increase plant performance [35,51].

## 4. Materials and Methods

### 4.1. Seed Collection and Germination in a Greenhouse

*Chenopodium quinoa* genotypes were obtained from the quinoa gene bank of the Universidad Nacional del Altiplano, Peru. Seeds of three genotypes of *Chenopodium quinoa,* with the codes BR2, R1, and Y2 (Table 2) were used for the experiment and then germinated in a greenhouse at the Xishuangbanna Tropical Botanical Garden, Chinese Academy of Science, China. The seeds were germinated in 10 March 2020. The daytime temperature was approximately from 22 to 26 °C and during the nighttime 18 to 24 °C. River sand was used as the soil medium for seed germination. After 8–10 days, healthy and similar-sized seedlings were transplanted into pots. The pots were watered every day to maintain soil moisture close to the water holding capacity.

### 4.2. Treatments

We conducted a three-factorial pot experiment including neighbour kinship (kin versus non-kin), nutrient treatments (low- versus high-nutrient), and root connectivity (connected versus disconnected). Seedlings were selected from seeds from three mother plants in each genotype. Seeds that were collected from the same genotype were considered kin, while seeds collected from different genotypes were considered non-kin. The size of each pot was 44 cm long, 28 cm wide, and 12 cm deep. Kin pots contained seedlings from a single genotype, whereas non-kin pots contained seedlings from all three different genotypes. In non-kin pots, seedlings of two different genotypes were interspersed. The genotypic identity of each seedling was known, allowing us to account for differences in growth potential between seedlings of the same and different genotypes in combination with kin and non-kin. The seedlings were planted 6 cm apart. For the low-nutrient treatment, we added 5 g fertilizer/kg (NPK 20:20:20), while for the high-nutrient treatment, we added 20 g fertilizer/kg nutrients (NPK 20:20:20) into the soil medium. We further included a root connectivity treatment to test whether direct root interaction was important for kin interaction. Connectivity was removed (“no connectivity” treatment) by pushing plastic slats down into the soil to the bottom of the pots between the seedlings. In the full connectivity treatment, no slats were pushed down. 

For each of the 8 combinations of the three variables with two levels (kin/non-kin, nutrients low/high, connection yes/no) there were 9 pots, yielding 72 pots in total. Each pot had 6 seedlings and the total seedling count was 432.

### 4.3. Morphological Traits and Biomass Measurement

The plants were harvested 70 days after planting. The following measurements were taken on each individual plant to know the growth performance of kin connected versus disconnected and non-kin connected versus disconnected plants under nutrient availability. We measured the total root length (cm, the sum of lengths of all individual roots) and specific root length (SRL, m g^−1^) of connected versus disconnected kin and non-kin pots. These root traits may describe the plant’s efficiency in searching for nutrient resources and understanding how the same individuals (kin) and different individuals (non-kin) interact (cooperate or compete) with each other belowground for the available resources. We also measured the stem length (cm, the length of the primary axis), dry masses (g, dried in an oven at 65 °C for 48 h) of the root, stem, and leaf organs. The dry mass data was then used to calculate the total dry mass (g) and organ mass fractions: leaf mass fraction (LMF, g leaf g^−1^ total mass), stem mass fraction (SMF, g stem g^−1^ total mass), and root mass fraction (RMF, g root g^−1^ total mass), specific stem length (SSL, m g^−1^), and leaf mass (LM, g). These traits might be better predictors indicating the competitive interaction between plants and plant organs because they are associated with soil prosperity and vegetation primary production. We further measured the leaf area ratio (cm^2^ g^−1^) and specific leaf area (cm^2^ g^−1^) because these traits are key among leaf traits and may tell us the ratio of the light-capturing surface of a leaf per unit investment of dry mass, as changes in leaf SLA reflect changes in the nutrient availability. We also measured this to know how much new leaf area to deploy for each unit of biomass produced when they grow in the combination of kin versus non-kin in response to nutrient availability.

### 4.4. Statistical Analysis

All statistical analyses were conducted using R 4.0.3. We used a linear mixed effects model to analyze the data, using the ‘lmer’ function of the *lme4* package [52] with kinship, fertility, and barrier as fixed effects in a full interaction model, with pot identity and genotype included as random effects. All response variables could be responding to kinship, barrier, and fertility, including total plant dry mass (TDM), shoot dry mass (SDM), organ mass fractions (LMF, SMF, RMF), organ efficiency parameters (SRL, SSL, and LM), leaf area ratio (LAR), and specific leaf area (SLA). Response variables were transformed as necessary to achieve approximate normality prior to regression analysis. All possible subset models of the fixed effects were compared on the basis of their AICc values, and the model with lowest AICc was considered to explain the response data best. This chosen model was then evaluated for explanatory power (estimated R^2^ values) using the (r.squaredGLMM) function of the *MuMIn* package [53]. Subsequently, pairwise Tukey HSD tests were conducted on the groups in the chosen model using the ‘glht’ function of the multcomp package [54]. We further conducted principal components analysis on the individual × traits data to understand which traits changed similarly across treatments, using the ‘rda’ function of the vegan package [55]. We then checked whether the first two axes of the pca were significantly related to each of the three treatments (kinship, barrier, fertility).

## 5. Conclusions

We conclude that quinoa plants accumulate greater biomass when competing for belowground resources with their close relatives than with their distant relatives. All plants independent of their kinship accumulated more biomass in high-nutrient soils. Consequently, we predict that quinoa plants will grow better in a field when cultivated with their close relatives (same cultivars/genotypes), rather than with distant relatives (different cultivars/genotypes). 

## Figures and Tables

**Table 1:** Summarized statistical results showing best linear mixed models selected for each response variable based on AICc comparisons (delta AICc < 2) of all subset models of the fixed effects (random effects were retained in all subset models). (delta AICc < 2). Transformations applied to normalize response data prior to regression are indicated. Coefficient estimates are conditional averages across selected models. Random effect variances and R^2^ estimates are taken from the maximum interaction model (R^2^m = marginal R^2^ value, shows the variance explained by fixed effects; R^2^c—condition R^2^ value, shows the variance explained by fixed plus random effects). “R”, “F”, and “B” represent relation, fertility, and barrier, respectively. Coefficient significance is indicated (* *p* < 0.05; ** *p* < 0.01, *** *p* < 0.001).

	Total Dry MASS (log)	Shoot Dry Mass (log)	Leaf Mass Fraction	Stem Mass Fraction	Root Mass Fraction (log)	Specific Stem Length (log)	Specific Root Length (log)	Average Leaf Mass (log)	Leaf Area Ratio	Specific Leaf Area
RANDOM EFFECTS (variance)										
PotID	0.20	0.22	0.07	0.08	0.27	0.25	0.35	0.22	0.75	28.69
Genotype	0.02	0.07	0.02	0.02	0.09	0.04	0.06	0.00	0.24	0.00
Residual	0.28	0.21	0.10	0.10	0.35	0.22	0.35	0.28	0.83	36.91
FIXED EFFECTS										
Intercept	−2.121	−2.031	0.571	0.547	−2.913	1.890	2.648	−5.239	4.232	125.8
Relation: non-kin	−0.344 **	−0.498 ***			−0.369 ***	−0.197 ***	−0.235 **	−0.343 ***	1.034 **	47.82 **
Fertility: low	−0.1384	−0.294 *	−0.094 ***	−0.099 ***	0.599 ***			−0.217 ***	−0.265	27.05
Barrier: dis	−0.418 ***	−0.555 ***			−0.193 **	0.204 **	0.318 ***	−0.444 ***	0.569	−6.912
R: non-kin x F: low	−0.198 *	−0.061							0.207	−37.02
R: non-kin x B: dis	0.253	0.544 **							−0.268	−1.146
F: low x B: dis	−0.511 ***	−0.198							−2.093 ***	−50.466 *
R: non-kin x F: low x B: dis	0.270	−0.185							−0.145	12.107
Explanatory power										
R^2^m	0.65	0.59	0.13	0.13	0.39	0.16	0.13	0.40	0.46	0.24
R^2^c	0.832	0.81	0.4	0.44	0.65	0.61	0.56	0.63	0.73	0.52

**Table 2:** Origin and characters of quinoa cultivars used in the experiment.

Genotype Local Code	Origin	Photoperiod Sensitivity	Morphological Characters
BR2	Peru (Puno)	Short day	White stem. Maturing medium-early to late. Yellow seeds; seed weight: 0.00349 g per seed.
R1	Peru (Casco)	Short–neutral day	Red stem and inflorescence, red young leaves, tolerant to frost and downy mildew. Early maturing. Black seeds; seed weight: 0.00272 g per seed.
Y2	Bolivia (southern altiplano)	Short day	Yellow stem. Panicle colored from white to yellow. Late maturing. Red seeds; seed weight: 0.00486 g per seed.

## Data Availability

Not applicable.
